# Early onset of efficacy in patients with functional and motility-related gastrointestinal disorders: A noninterventional study with Iberogast®

**DOI:** 10.1007/s10354-017-0578-y

**Published:** 2017-07-25

**Authors:** Richard Raedsch, Bettina Vinson, Bertram Ottillinger, Gerald Holtmann

**Affiliations:** 1 St. Josefs-Hospital, Beethovenstraße 20, 65189 Wiesbaden, Germany; 2 Steigerwald Arzneimittelwerk GmbH, Havelstraße 5, 64295 Darmstadt, Germany; 3 Ottillinger Life Sciences, Föhrenstraße 12, 85649 Hofolding, Germany; 4 Faculty of Health and Behavioural Sciences and Faculty of Medicine & Biomedical Sciences, Princess Alexandra Hospital, University of Queensland, 37 Kent Street, Woolloongabba/Brisbane, Australia

**Keywords:** Functional dyspepsia, Irritable bowel syndrome, Iberogast®, Iberis amara, Early onset, Funktionelle Dyspepsie, Reizdarm, Iberogast®, Iberis amara, Frühzeitiger Beginn

## Abstract

STW 5 (Iberogast®; Steigerwald Arzneimittelwerk GmbH, Darmstadt, Germany) contains nine plant extracts and possesses well-documented overall efficacy in functional gastrointestinal disorders (FGID). Little is known about the onset of symptom relief. Twenty-nine centers in Germany recruited 272 patients with established FGID. These patients were treated with STW 5 for approximately 3 weeks in this noninterventional study. Patients assessed the severity of their gastrointestinal complaints before and at defined times after the intake of STW 5 (10 cm visual analogue scale; VAS). Fifteen minutes after the first dose, the severity of gastrointestinal complaints had decreased by 1.4 cm (mean; initial mean: 5.2 of 10 cm). After 1 h, more than 90% of the maximum effect of 3.2 cm on the 10 cm VAS had been reached. Most patients with symptoms experienced a marked improvement within 5, 15 or 30 min of taking STW 5. Absolute improvements were larger in patients with more pronounced baseline complaints. Subgroups with upper (80% of the study population) and lower FGID (20%) did not present major differences. Neither did subgroups by age and duration of complaints. Treatment with STW 5 resulted in rapid improvement of symptoms.

## Zusammenfassung

STW 5 (Iberogast®; Steigerwald Arzneimittelwerk GmbH, Darmstadt, Deutschland) besteht aus neun pflanzlichen Wirkstoffen und besitzt eine gut dokumentierte Wirksamkeit bei funktionellen gastrointestinalen Erkrankungen (FGID). Über die Zeit bis zum Wirkungseintritt lagen nur wenige Daten vor. Neunundzwanzig Zentren in Deutschland rekrutierten 272 Patienten mit etablierter FGID, die in dieser nichtinterventionellen Studie über ca. 3 Wochen mit STW 5 behandelt wurden. Die Patienten bewerteten den Schweregrad ihrer gastrointestinalen Beschwerden vor und zu definierten Zeitpunkten nach der Einnahme von STW 5 (visuelle 10-cm-Analogskala; VAS). Der Schweregrad der Beschwerden hatte 15 min nach der ersten Dosis um 1,4 cm abgenommen (Mittelwert; Ausgangswert 5,2 von 10 cm). Nach 1 h waren mehr als 90 % der maximalen Wirkstärke von 3,2 cm auf der 10-cm-VAS erreicht. Die Mehrheit der Patienten berichtete über eine ausgeprägte Verbesserung innerhalb von 5, 15 oder 30 min nach der Einnahme. Patienten mit ausgeprägteren Beschwerden zeigten eine größere absolute Verbesserung. Untergruppen mit FGID des oberen (80 % der Population) bzw. unteren Magen-Darm-Trakts (20 %) zeigten keine größeren Unterschiede, ebenso wenig wie Subgruppen nach Alter und Dauer der Beschwerden vor Behandlung. Die Behandlung mit STW 5 führte zu einer schnellen Verbesserung von Symptomen.

## Introduction

**Fig. 1:**
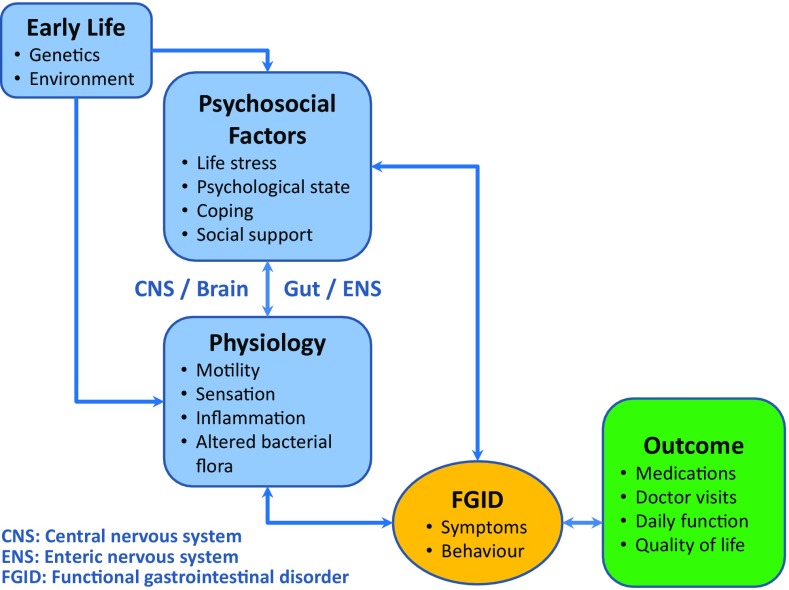
Model of functional gastrointestinal disorders [5]

Functional gastrointestinal disorders (FGID) like functional dyspepsia or irritable bowel syndrome are frequent reasons for patient visits to doctors. Depending on their definition, the prevalence of FGIDs in Western countries ranges from 10% to more than 20% [1–3]. In these patients, diagnostic work-up in the clinical setting does not reveal structural or biochemical abnormalities that can explain the respective symptoms. FGIDs are understood as multifactorial biopsychological phenomena [4]. Thus symptoms are now attributed to a complex interaction of physiologic and psychosocial factors, which are interlinked through the “brain–gut axis” (Fig. 1; [5]). Since 2006, clinicians and also clinical trials most frequently use the Rome III criteria to standardize diagnosis of FGIDs. These criteria classify FGIDs into several categories according to the location of symptoms, the predominant symptoms and the patient’s age. It is acknowledged that complaints may overlap, i. e. that patients may present symptoms of more than one category. In adults, six major domains have been defined: esophageal (category A); gastroduodenal (category B); bowel disorders (category C); functional abdominal pain syndrome (category D); biliary (category E); and anorectal disorders (category F) [5]. This study focused on functional and motility related disorders, which are allocated to the gastroduodenal and bowel (intestinal) domain (categories B and C).

The therapy for FGIDs includes psychotherapy, dietary recommendations, relaxation and stress management as well as pharmacologic interventions like proton pump inhibitors, H2 antagonists, herbal medicines, prokinetic drugs, laxatives and antacids [1–3]. Most drugs treat only one symptom category at a time, so that patients with overlapping and variable complaints may require more than one drug [6].

The herbal combination medicine STW 5 (Iberogast®; Steigerwald Arzneimittelwerk GmbH, Darmstadt, Germany) was developed to address these overlapping and variable complaints combining defined plant extracts as Active Pharmaceutical Ingredients (APIs). STW 5 contains alcoholic extracts of *Iberis amara totalis recens, Angelicae radix, Cardui mariae fructus, Chelidonii herba, Liquiritiae radix, Matricariae flos, Melissae folium, Carvi fructus* and *Menthae piperitae* *folium* and has been used for the therapy of FGIDs for more than five decades. The extracts exert well-defined pharmacological effects on different regions of the gastrointestinal tract. Thus, they influence gastroduodenal as well as intestinal symptoms. The components are the subject of Herbal Drug Monographs [20–27] and of studies performed with the defined extracts of STW 5 [19, 28–32, 41]. They regulate motility by acting on the gastrointestinal smooth muscles in an area-specific manner, stimulate gastric secretion and exert spasmolytic, choleretic, anti-inflammatory and carminative effects.

Indeed, numerous clinical studies of different designs (placebo-controlled [11, 13, 14, 16]; vs. active comparator [15]; observational [17, 18]) have been conducted with STW 5 and compiled in reviews [10, 41] and a meta-analysis [12] to assess its efficacy in the treatment of FGIDs.

However, while the overall efficacy is well documented in clinical trials, very little is known about the onset of symptom relief. This is a highly relevant question in the clinical setting and has been studied for other compounds and other gastrointestinal indications like gastroesophageal reflux disease, heartburn and ulcerative colitis [33–38]. To close this gap for STW 5, we conducted a noninterventional (observational) study (NIS) to assess the time to onset of symptom improvement after initiation of therapy with STW 5.

## Material and methods

### Participants and sample size

Thirty centres in Germany, mostly general practitioners, were invited to participate in this study. Investigators recruited male or female patients over 18 years of age with an established diagnosis of FGID (categories B and C according to the Rome III criteria [39, 40]). Physicians had to take care that all patients recruited were eligible according to the STW 5 summary of product characteristics (SmPC). Since this study also aimed to provide additional information on drug safety, the sample size was estimated on the basis of the power to detect adverse events. The aim was to detect adverse events with a true incidence of 1% with a probability of at least 95%, which required 300 patients.

### Therapy

In most countries STW 5 is approved for the treatment of functional and motility related disorders of the gastrointestinal tract, including functional dyspepsia and irritable bowel syndrome. The symptoms generally comprise gastric pain, heartburn, abdominal fullness, gastric and intestinal spasms and/or nausea. In line with the SmPC, the recommended dosage of STW 5 (Iberogast®) in this study was 20 drops three times a day (t.i.d.), taken in liquid before or with meals.

There were no restrictions with regard to other medications but all applied medications other than STW 5 had to be documented.

### Study objectives, schedule and outcomes

The primary objective of this noninterventional study was to assess the time to onset of symptom improvement after each STW 5 dose during the first 8 days of therapy. Overall, each patient was followed up for approximately 3 weeks, with investigator visits at start and end of therapeutic intervention. At the first visit, the investigators documented patient medical history, any diagnostic gastrointestinal measures including duration of gastrointestinal disorders and applied therapeutic interventions, current gastrointestinal symptoms and concomitant diseases.

After the first dose of STW 5 on day 1, the patients assessed the severity of their complaints on a 10 cm visual analogue scale (VAS) before and 1, 5, 15, 30, 60 and 120 min after the dose. The ends of the scale were marked with “no complaints” and “severe complaints”. Furthermore, after every dose of STW 5 during the following 8 days, the patients documented the time to marked improvement of their functional gastrointestinal complaints in a patient diary (seven categories: within 1 min, 5 min, 15 min, 30 min, 1 h, 2 h or longer than 2 h).

As a secondary objective, the investigators assessed changes of the gastrointestinal symptoms of patients with the GIS (Gastrointestinal Symptom Profile) score from the start of therapy to the end of therapy at visit 2. The score was validated and employed in various STW 5 studies of functional dyspepsia [7, 8]. The GIS is an interview instrument including 10 symptoms specific for functional dyspepsia (Table 1). Each symptom is evaluated on a 5-step Likert scale, ranging from 0 = none to 4 = very pronounced. The GIS sum score ranges from 0 to 40. The GIS was evaluated at the first and last study visit. In addition, during the last visit investigators and patients assessed the change of gastrointestinal symptoms versus the first visit on 4‑step Likert scales.

**Table 1:** Gastrointestinal symptom (GIS) profile

1. Nausea
2. Sickness
3. Vomiting
4. Bloating
5. Abdominal cramps
6. Early satiety
7. Acidic eructation/heartburn
8. Loss of appetite
9. Retrosternal discomfort
10. Epigastric pain/upper abdominal pain

Safety endpoints were documented during the entire study period and included adverse events, adverse drug reactions and drug interactions. At the end of the study and if patients discontinued the therapy with STW 5 prematurely, investigators were requested to ask for and document adverse events, reasons for discontinuation and new initiated therapies.

### Legal aspects and quality control

Following the principles of noninterventional (observational) studies, the investigators were free to follow usual best practice when treating their patients. The study protocol standardised documentation, but did not interfere with therapy or visit schedules.

The study was performed in accordance with the applicable regulatory requirements in Germany, i. e. § 67 (6) of the German Drug Law, including notifications to the relevant authority, to the national association of panel doctors and to the association of statutory health funds. Representatives of the sponsor and monitors of a contract research organisation contacted the investigators before the study to explain the documentation of parameters and adverse events. During and after the study, the participating centres were contacted and the documentation forms were checked for completeness and plausibility at site closure.

### Data management and statistics

Data were entered into a database and analysed utilising SAS® (Statistical Analysis Software). The data were checked electronically for plausibility and validity. Concomitant medication was coded using the WHO (World Health Organization) drug reference list, diagnoses, concomitant diseases and adverse events were coded in MedDRA (Medical Dictionary for Regulatory Activities).

The statistical analyses employed the SAS® Version 9.1.3 software package. Data were tabulated using descriptive statistics appropriate to the scale. Complex information was displayed graphically. Descriptive two-sided 95% confidence intervals were calculated where adequate. Analyses of the GIS score, of the severity of complaints (VAS), of the global assessments and of safety endpoints were furthermore performed for the indication subgroups functional gastro*duodenal *disorders (functional dyspepsia, functional eructation, nausea and vomiting) and functional gastro*intestinal* disorders (irritable bowel syndrome, functional distension, functional constipation, functional diarrhoea). The improvement of gastrointestinal complaints was furthermore analysed in subgroups defined by baseline severity, age, and duration of complaints, and by investigational site. All subgroup analyses were exploratory and no 95% confidence intervals or *p*-values were calculated for differences between subgroups. The *p*-values calculated for influence of baseline severity, age and duration of complaints are descriptive and do not indicate confirmatory analyses.

## Results

### Participants

Between July 2008 and April 2010, potential sites received 300 documentation forms. Of 30 sites invited to participate, 29 active sites recruited and documented 272 patients treated with STW 5. The statistical analyses considered all patients. Following consultations with the Coordinating Investigator of this study (RR), 9 patients (3.3%) violating the lower age limit of 18 years were not excluded from the analyses, as they contribute information to the real-world population treated with STW 5. Furthermore, the youngest patients were 5 years of age and thus able to explain themselves. They were within the lower border of the Rome III criteria’s validity limit (4 years) [42].

The lower number of patients compared to the originally planned sample size (*n* = 272 vs. *n* = 300) increased the true incidence of adverse events detectable with a power of 95% from 1.0% to 1.1%.

### Demography and baseline data

Relevant baseline data are summarised in Table 2. Most patients suffered from functional gastroduodenal complaints, i. e. symptoms affecting predominantly the upper abdomen (80%). Almost 40% had a disease history of 6 months or longer, satisfying the formal Rome III diagnostic criteria for functional gastrointestinal disorders. The current episode for which patients were treated and documented in this study had lasted for 3 weeks or less in over 70% of patients. Fifty-four percent of patients were female. Almost every second patient reported concomitant diseases (49%), most frequently arterial hypertension (35%) and diabetes mellitus (12%). Twenty percent of the sample were 65 years or older and 46% were not working or caring for the household. Twenty two percent of the study population had one or more sick leaves in the preceding 3 months because of functional gastrointestinal complaints. One third of patients had visited a physician for three or more times during the preceding 3 months because of their complaints, another 38% at least once. These data indicate a significant disease burden.

**Table 2:** Baseline data

Parameter	*n* (%) or mean ± SD
Evaluable patients	272 (100)
**Subgroup of functional gastrointestinal disorder**	
Gastroduodenal	218 (80)
Intestinal	54 (20)
Female	146 (54)
Male	126 (46)
**Age**	49 ± 18 years
Age range	5–92 years
5 to <18 years	9 (3)
18 to <50 years	125 (46)
50 to <65 years	84 (31)
≥65 years	54 (20)
**BMI**	22.5 ± 4.0 kg/m^2^
BMI range	8.0–38.3 kg/m^2^
Employed/working	147 (54)
Not employed/not working	125 (46)
Family history of functional GI disorders	121 (44)
**Duration of GI complaints**	
3 months	108 (40)
3 to <6 months	60 (22)
6–12 months	39 (14)
> 12 months	65 (24)
**Duration of current episode**	
1 week	81 (30)
1–3 weeks	117 (43)
> 3 weeks	74 (27)
**Concomitant diseases**	133 (49)
Arterial hypertension	96 (35)
Diabetes mellitus	32 (12)
Coronary heart disease/arteriosclerosis	26 (10)

*GI* gastrointestinal, *BMI* body mass index, *SD* standard deviation

Three of 4 patients (*n *= 201; 74%) had received medication for earlier episodes of functional gastrointestinal complaints. Most frequently these were proton pump inhibitors (49% of patients with medication), antacids (42%), prokinetic drugs (36%) and H2 blockers (26%). Only 22 patients (8% of the entire study population) reported previous experience with Iberogast®.

### Therapy

Patients were documented for 20 ± 7.5 days (mean ± SD; median: 21 days), which reflects the intended study duration of 21 days. Overall, 69 patients (25%) discontinued STW 5 prematurely. The most frequent reasons were complete relief of symptoms (*n *= 45; 65%) and patient’s request (*n *= 12; 17%). Only 7 patients discontinued treatment for lack of efficacy (10% of early discontinuations). No patient discontinued treatment with STW 5 because of adverse events or adverse drug reactions.

After the end of this study, 163 patients (60%) required continued therapy. Most of these patients continued STW 5 as monotherapy (*n *= 145; 89% of continued therapies) or in combination with another drug (*n *= 4; 2%). Only 14 patients started therapy with other drugs (9%).

### Onset of effectiveness – visual analogue scale (first dose)

**Fig. 2:**
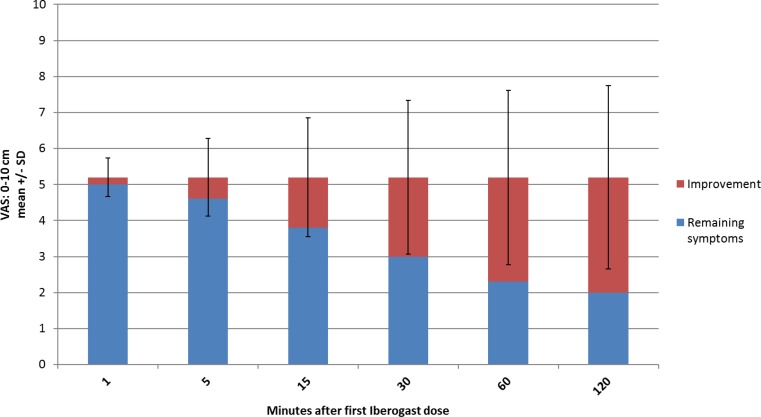
Improvement of complaints after the first Iberogast® dose (0–10 cm VAS)

At the first visit and before the first intake of STW 5, patients judged the severity of gastrointestinal complaints at 5.2 ± 3.2 cm (Mean ± standard deviation [SD]) on the 10 cm VAS (median: 6.3). After the intake of medication, the severity decreased continuously over the following 2 h. The largest improvements per interval appeared already within the first and the second 15 min intervals (see Fig. 2). As early as 15 min after the dose, the severity of complaints had decreased by more than 1 cm (1.4 ± 1.7 cm, Mean ± SD), which is an amount clearly noticeable and thus clinically relevant. After 1 h, more than 90% of the maximum effect (a decrease by 3.2 ± 2.6 cm, determined after 2 h) had been reached.

### Time to marked improvement (patient diary)

This parameter was evaluated on the first eight study days. On all days, the majority of patients with symptoms experienced a marked improvement within 30 min of taking STW 5. The onset of effectiveness was similar for the morning, noon and evening doses. On days 1 and 2, the categories chosen by most patients were response within 15, 30 or 60 min after the dose. Over time, a shift towards an earlier onset of effectiveness could be seen, with the most frequently chosen time categories being 5, 15 or 30 min after the dose.

**Fig. 3:**
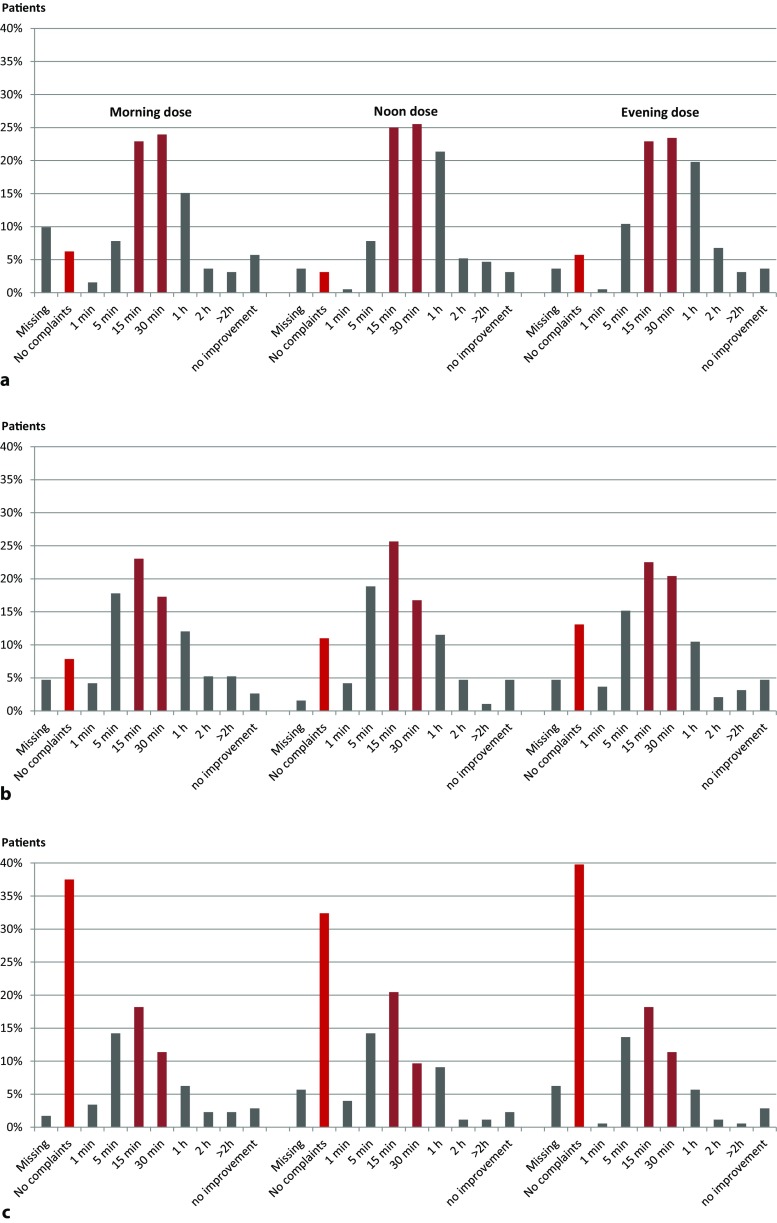
**a–c** Proportion of patients who report time to onset of marked imrpovement after a single dose of Iberogast® at morning, noon and evening for day 1 (**a**), day 4 (**b**) and day 8 (**c**)

From days 3 and 4, up to 40% of patients became completely free of symptoms, consistent with a profound improvement of their complaints. For methodological reasons, these patients could not choose temporal response categories. Fig. 3 illustrates the pattern of patient responses and their change from day 1 over day 4 to day 8.

### GIS and global assessments

**Fig. 4:**
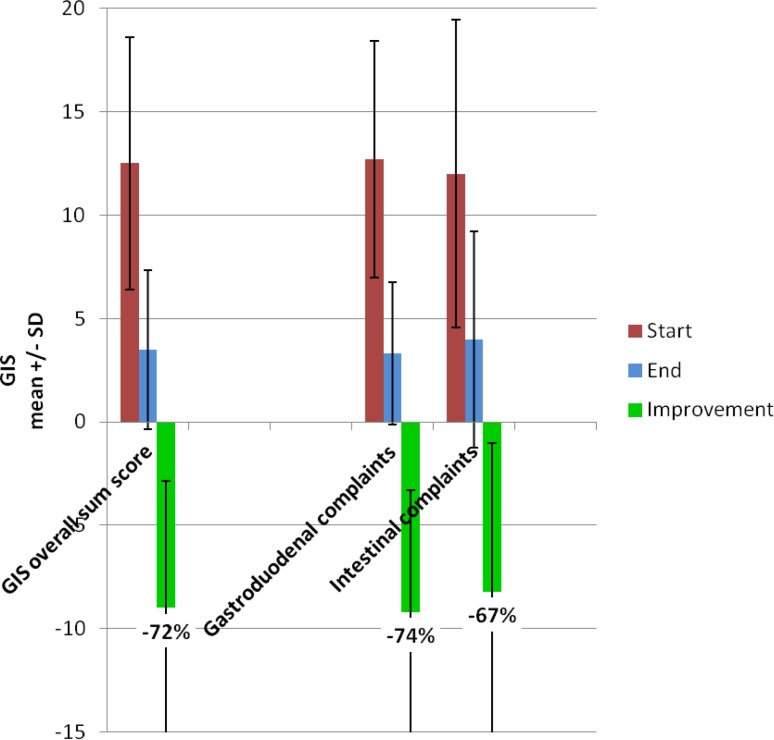
Gastrointestinal symptom profile (GIS) at study start and end

The mean GIS score at the start of the study was 12.5 ± 6.1 points (median: 12). Over the study period, the mean GIS decreased by 9.0 ± 6.2 points to 3.5 ± 3.8 points, corresponding to a mean relative reduction of 72% versus baseline (see Fig. 4). Patients and investigators rated the global treatment effect similarly, with 74 to 79% ratings indicating complete or marked improvement vs. baseline (Table 3). Patients’ and investigators’ global assessments correlated highly with improvements of the GIS score (explorative *p* for Spearman’s rank correlation: *p* < 0.0001).

**Table 3:** Global assessment of the treatment effect by investigators and patients

Global assessment of the treatment effect	Investigator		Patient	
	*n*	(%)	*n*	(%)
**Missing (all)**	9	3	12	4
Gastroduodenal (*n* = 218)	6	3	11	5
Intestinal (*n* = 54)	3	6	1	2
**Complaints completely improved (all)**	69	25	79	29
Gastroduodenal	56	26	63	29
Intestinal	13	24	16	30
**Complaints markedly improved (all)**	147	54	121	44
Gastroduodenal	124	57	101	46
Intestinal	23	43	20	37
**Complaints somewhat improved (all)**	41	15	51	19
Gastroduodenal	29	13	38	17
Intestinal	12	22	13	24
**Complaints not improved (all)**	6	2	9	3
Gastroduodenal	3	1	5	2
Intestinal	3	6	4	7

### Tolerability

Two of the 272 patients treated with STW 5 (0.7%) reported four adverse events (hyperuricaemia, tick bite, pharyngitis, lumbago). These events were nonserious and considered unrelated to Iberogast®. The investigators reported no suspected interactions between study medication and other drugs.

### Subgroup analyses

Investigators classified the 80% of study population as predominantly suffering from functional gastro*duodenal* complaints, whereas 20% (*n *= 54) presented predominantly other functional gastro*intestinal *complaints.

*Therapy:* Patients with gastroduodenal complaints were treated for a similar period of time as patients with other gastrointestinal complaints (21 ± 7.3 days vs. 19 ± 7.9 days). In the subgroup with gastroduodenal complaints, 20% of patients discontinued therapy prematurely, mostly due to complete relief of symptoms (73% of discontinuations) or patient’s request (11%). In the subgroup with gastrointestinal complaints the overall rate of premature discontinuations was higher (46%). Complete relief of symptoms was the most frequent reason also in this subgroup (52%), followed by patient’s request (28%). The proportions of discontinuations due to a lack of efficacy were similar in both subgroups (gastroduodenal: 11%; gastrointestinal: 8%). After the end of the study, fewer patients in the subgroup with intestinal complaints required further therapy than in the subgroup with gastroduodenal complaints (48% vs. 63%). Of the patients requiring continued therapy, most continued STW 5 (85% and 93%, respectively).

*Onset of effectiveness:* Patients with gastrointestinal complaints had more pronounced baseline symptoms than patients with gastroduodenal complaints as measured with the VAS on day 1 (6.1 ± 3.0 cm vs. 4.9 ± 3.3 cm; Mean ± SD), but also a somewhat higher improvement after 2 h (3.7 ± 2.7 cm vs. 3.0 ± 2.5 cm; Mean ± SD). Both groups showed a clinically relevant improvement as early as 15 min post dose (1.7 ± 2.1 cm and 1.3 ± 1.5 cm, respectively; Mean ± SD). In both groups, at least 90% of the maximum effect (determined after 2 h) had been reached already after 1 h. The time to marked improvement, assessed by the patients over 8 days after each dose, as well as the proportion of patients achieving complete freedom of symptoms, were similar in both subgroups.

*GIS and global assessments:* The GIS score improved in patients with gastroduodenal and in patients with intestinal complaints to a similar extent. There were quantitative, but no qualitative differences between these groups (Fig. 4). The global assessments showed minor differences between the subgroups, but in both subgroups, the proportion of patients with complete improvement was almost identical (Table 3).

*Improvement of gastrointestinal complaints by baseline severity:* The influence of baseline severity on improvement of symptoms was analysed by quartiles (1^st^ quartile: 0.9 cm on the 10 cm VAS; 2^nd^: 6.2 cm; 3^rd^: 7.8 cm; 4^th^: 10.0 cm). Patients in the 1^st^ quartile showed a lower absolute improvement than patients in the 2^nd^, 3^rd^ or 4^th^ quartile (−0.55 cm 120 min after intake of STW 5 vs. −3.75 cm; −4.69 cm; and −6.01 cm; adjusted mean estimates). This pattern was apparent for all times after intake and a repeated measurement analysis of VAS absolute change to baseline including the factors time, baseline VAS and baseline VAS by time interaction showed a significant effect of baseline VAS (*p *< 0.0001) as well as a significant time by baseline VAS effect. Therefore, the treatment effect was higher for patients with higher baseline VAS values.

*Improvement of gastrointestinal symptoms by age group:* Age was categorized as 0 to <18 years (*n *= 5), 18 to <50 years (*n *= 80), 50 to <65 years (*n *= 60) and ≥65 years (*n *= 41). The youngest age group showed the highest absolute improvement of symptoms 120 min after intake of STW 5, followed by the age groups 18 to <50 years, ≥65 years, and 50 to <65 years (−4.85 cm vs. −3.44 cm vs. −3.00 cm vs. −2.80 cm; adjusted mean estimates). This pattern was apparent for all times after intake. Age seemed to be a predictive factor (*p *= 0.0242); however, the youngest age group driving this effect was very small (*n *= 5) and the effect in the other age groups comparatively homogenous.

*Improvement of gastrointestinal complaints by duration of complaints:* The duration of gastrointestinal complaints was categorized as <3 months (*n *= 74), 3 to <6 months (*n *= 41), 6–12 months (*n *= 24) and >12 months (*n *= 47). The group with the shortest duration of complaints before the start of therapy with STW 5 showed the highest absolute improvement of symptoms 120 min after intake of STW 5, followed by the groups with a duration of complaints of >12 months and 3 to <6 (which were barely discernible) and the group with a duration of complaints of 6–12 months (−3.60 cm vs. −3.05 cm vs. −3.03 cm vs. −2.33 cm; adjusted mean estimates). This pattern was apparent for all times after intake. Though numerically conspicuous, the duration of GI complaints did not seem to be a predictive factor (*p *= 0.3122).

Improvement of gastrointestinal complaints by investigational site: Differences between sites in improvement of gastrointestinal complaints coincided with baseline complaints, in that sites with less severely affected patients found less pronounced improvements. No other patterns appeared.

## Discussion

This noninterventional study confirmed that STW 5 improved symptoms of upper and lower functional gastrointestinal disorders (FGIDs). The gastrointestinal symptom score GIS, validated for FGIDs, improved by more than 70% versus baseline over the treatment period of 3 weeks. Investigators’ and patients’ global assessments of treatment effect were comparably favourable, correlated significantly with changes of the GIS and thus confirmed the internal validity of this study.

Noninterventional (observational) studies are useful to validate the data regarding efficiency and safety from controlled clinical studies and test whether these findings can be extrapolated to daily medical practice. Controlled clinical studies are standardised scientific experiments, where patients are carefully selected according to very strict inclusion criteria. As a consequence, concomitant diseases are commonly excluded as possible confounding factors. Thus, results of such trials may not be relevant and applicable to the average patient who would not qualify for a clinical study. Therefore it is highly relevant for clinical practice that STW 5 showed effectiveness in this noninterventional study similar to that seen in controlled clinical studies versus placebo and/or active drug [10–18, 41]. Other noninterventional studies and retrospective database surveillances came to comparable conclusions [10, 41]. While this was not a placebo-controlled trial, the data are aligned with the results of previous placebo-controlled trials and the data from the noninterventional observational study suggest that 40% of patients treated experience substantial improvement (absence of symptoms) after 3 to 4 days of treatment. Thus, in the clinical routine 2 out of 3 patients will experience at least noticeable relief of symptoms during treatment with STW 5.

A substantial proportion of the patient sample suffered from concomitant diseases (49%), including arterial hypertension and diabetes mellitus. In these patients, the overall response rate was not different and there was no increase of adverse outcomes.

The clinical effect of STW 5 was overall comparable in patients with symptoms preferably referred to the upper or lower gut. This applied especially to the onset of symptom control as measured with the GIS. The GIS assesses ten symptoms formally validated only for patients with functional dyspepsia. Nevertheless, it showed comparable effects also in the patients with functional intestinal disorders. This underlines its usefulness also in this population and indicates that patients with lower FGIDs or overlapping complaints recognize their symptoms in the GIS. The onset of effectiveness was determined by one global question not related to distinct gastrointestinal symptoms. The congruence of this endpoint with other endpoints substantiates the patients’ ability to subjectively identify symptoms they consider important and requiring therapy.

Minor differences between the subgroups with upper and lower FGIDs regarding the overall assessments, the discontinuations of therapy and the patients requiring continued therapy after the study showed no clear profile in favor of one indication subgroup. This is important because patients may suffer from upper and from lower functional gastrointestinal complaints at the same time or in successive episodes and because symptoms and the pathophysiology may overlap. Thus, STW 5 with a variety of active components is a suitable treatment option also for these patients, and not only for patients with clearly differentiated FGDs [10, 41–44].

Specific subgroup analyses found a larger absolute improvement of gastrointestinal complaints in patients with a higher baseline severity. This is reassuring in that STW 5 is a treatment option also for the patients requiring therapy most. Further subgroup analyses showed that effectiveness was largely independent of the duration of complaints, investigational site and age. The large improvement in younger patients <18 years of age, though highly satisfactory, should be treated with caution: the patient group in questions was small and possible confounded, as these patients may have been assisted by parents in documenting their assessments.

This study demonstrates rapid improvement of symptoms in patients treated with STW 5. An improvement of symptoms was noticeable within 15 min after the first dose, and the majority of patients experienced a relevant improvement within 15–30 min after each STW 5 dose. There was a tendency towards an earlier improvement as therapy continued. Thus there is no evidence for habituation to the treatment effect.

In this noninterventional (observational) study in patients with functional and motility-related gastrointestinal complaints, treatment with STW 5 resulted in a fast improvement of symptoms and moreover showed a treatment effect comparable to that observed in controlled clinical studies. Forty percent of patients became completely free of symptoms after 3 or 4 days of treatment.

The results were comparable in the subgroups with upper and with lower functional gastrointestinal complaints. Thus, in this noninterventional study STW5 was confirmed by patients as relevantly fast acting medication for upper and lower gastrointestinal complaints within FGIDs.
